# Age-stratified survival and competing mortality in adult acute lymphoblastic leukemia and lymphoblastic lymphoma: a population-based competing-risk and restricted mean survival analysis

**DOI:** 10.3389/fonc.2026.1913564

**Published:** 2026-10-12

**Authors:** Zhe Li, Jianglin Yu

**Affiliations:** 1 Department of Hematology, Guizhou Provincial People’s Hospital, Guiyang, Guizhou, China; 2 The First Clinical Medical College, Zunyi Medical University, Zunyi, Guizhou, China

**Keywords:** acute lymphoblastic leukemia, age stratification, competing risk, lymphoblastic lymphoma, population-based study, restricted mean survival time, SEER

## Abstract

**Background:**

Survival differences by age are well recognized in adult acute lymphoblastic leukemia/lymphoma (ALL/LBL), but their timing, competing-mortality structure, and behavior across recent diagnosis periods remain less clear.

**Methods:**

This retrospective SEER cohort included adults diagnosed during 2010-2023, excluding Burkitt leukemia/lymphoma. We estimated overall survival (OS), 3-year restricted mean survival time (RMST), and cumulative incidence of ALL/LBL-attributable and other/unknown-cause death. Cox, Fine-Gray, spline, and exploratory time-split models assessed age associations.

**Results:**

Among 9,491 patients, 4,618 died: 3,828 deaths were attributed to ALL/LBL and 790 to other or unknown causes. Three-year RMST was 1.89, 2.10, and 2.14 years in 2010-2014, 2015-2019, and 2020-2023, respectively; the recent-period difference was 0.044 years (95% CI -0.016 to 0.104; P = 0.150). Median follow-up in 2020–2023 was 22 months. Compared with age 18–39 years, the adjusted overall-mortality HR for age >=75 years was 6.258 (95% CI 5.627-6.960). Because proportional hazards were violated, this HR should be read as a summary association. The exploratory HR was 8.201 (95% CI 7.224-9.311) during the first year and 3.496 (95% CI 2.885-4.236) thereafter. At 3 years, cumulative incidence in patients aged >=75 years was 70.4% for ALL/LBL-attributable death by registry attribution and 12.8% for other/unknown-cause death. The spline test supported departure from linearity (P<0.001); the age-by-period interaction was not significant (P = 0.182), whereas an exploratory age-by-subtype interaction was nominally significant (P = 0.024, unadjusted for multiplicity).

**Conclusion:**

Older age was associated with higher mortality, especially in the first year after diagnosis. ALL/LBL-attributable death predominated by registry attribution even in the oldest group. The 3-year comparison did not establish a survival difference between 2015–2019 and 2020-2023; limited follow-up and residual case-mix differences preclude concluding that progress has stopped.

## Introduction

Adult acute lymphoblastic leukemia (ALL) comprises biologically distinct diseases with widely different prognoses (1, 2). Risk assessment now integrates clinical features, disease genetics, and treatment response; management has also expanded to include targeted agents and immunotherapy (3–5). Even so, older adults continue to fare worse and often tolerate intensive therapy less well (6, 7). Population-based studies have reported survival gains, but the age gap has not disappeared (8, 9).

Recent registry studies have examined adolescent and young-adult ALL/LBL survival and outcomes by reported Philadelphia-chromosome classification (10, 11). Cross-period interpretation remains difficult for two reasons. Overall survival combines deaths attributed to ALL/LBL with deaths from competing causes, and the most recent cohorts contribute less long-term follow-up. Cumulative incidence and Fine-Gray analyses separate the mortality endpoints (12, 13). RMST, in turn, compares the area under survival curves within a shared time horizon and does not require proportional hazards (14, 15).

Age categories are clinically convenient, but they can hide meaningful variation within groups. A single age-associated HR may also blur the distinction between early and later follow-up. These issues are especially relevant for older adults, whose disease biology, treatment tolerance, and competing mortality differ from those of younger patients (4, 6, 7).

Using SEER data, we examined age-related survival and competing mortality in adult ALL/LBL diagnosed during 2010-2023. The analysis extends population-level evidence into the early 2020–2023 treatment era, quantifies age-stratified competing mortality, and compares diagnosis periods with common-horizon RMST rather than immature long-term survival estimates. We also evaluated nonlinear age associations, changes over follow-up, and heterogeneity across histologic subtypes.

## Materials and methods

### Data source and study population

This retrospective cohort study used SEER Research Plus Data, 17 Registries, November 2025 Submission (2000-2023), extracted with SEER*Stat version 9.0.43 (16). The export included ICD-O-3 codes 9811–9819 and 9837 for B- and T-lineage ALL/LBL, as well as code 9826 for Burkitt leukemia/lymphoma. Burkitt leukemia/lymphoma is a mature B-cell neoplasm, so it was excluded from the primary cohort and re-included only in a sensitivity analysis. In this manuscript, lymphoblastic lymphoma refers to the leukemia/lymphoma combined codes captured in the export; separate nodal LBL codes 9728 and 9729 were not part of the analytic definition. The findings therefore apply to this code-defined cohort, not to every possible ALL/LBL coding category.

Eligible patients were aged >=18 years, diagnosed during 2010-2023, had a first primary malignancy (“one primary only” or “1st of 2 or more primaries”), and had usable survival information. Of 9,844 exported records, 32 were excluded because complete dates indicated 0 days of survival, 69 lacked usable nonnegative survival time, and 252 had Burkitt histology. The final cohort included 9,491 patients. Unknown race or marital status did not lead to exclusion in the primary analysis. **Supplementary Table S2** gives the sequential counts. Study size was determined by all eligible records in the export; no prospective sample-size calculation was performed.

### Variable definitions

Analyzed variables included age, sex, race, diagnosis year, marital status, histologic subtype, treatment records, and survival outcomes. Age was grouped as 18-39, 40-59, 60-74, and >=75 years for clinical interpretability. These cut-points were fixed in the analysis code and were not optimized against outcomes; prospective prespecification was not documented. They should therefore be read as pragmatic categories, not validated biological thresholds. Diagnosis periods were 2010-2014, 2015-2019, and 2020-2023.

Histology was classified as B-ALL/LBL NOS (9811), B-ALL/LBL with BCR-ABL1 (9812), other genetically defined B-ALL/LBL (9813-9819), or T-ALL/LBL (9837). These are registry-coded categories. The NOS group does not establish negative BCR-ABL1 status, and the export lacks complete patient-level cytogenetic and molecular characterization. B-lineage analyses combined codes 9811-9819; T-lineage analyses used 9837.

Marital status was grouped as married, single, divorced/separated/widowed, or unknown. Chemotherapy was recorded as yes versus no/unknown. Radiation was coded as documented administration versus none/unknown, with refusal and a recommendation of uncertain administration included in the latter category. Neither combined category establishes that all patients were untreated. Unknown race and marital status were retained as explicit categories; no multiple imputation was performed.

### Endpoints

OS was measured from diagnosis to death from any cause; patients alive at last contact were censored then. ALL/LBL-attributable death was defined by the SEER classification “Dead (attributable to this cancer dx),” referring here to the index ALL/LBL diagnosis. Remaining deaths formed an other/unknown-cause-of-death (COD) endpoint, including 106 deaths with missing or unknown COD. A sensitivity analysis censored those 106 deaths at their recorded times.

Cause-specific hazards describe the instantaneous event rate among patients still alive. Fine-Gray subdistribution hazards use a modified risk set that retains patients with competing events; therefore, an SHR is not a cause-specific HR and is not a ratio of death probabilities (12, 13). Cumulative incidence estimates provide absolute probabilities by a specified time. Percentages at last follow-up are reported separately as crude proportions of all patients in each age group.

### Statistical analysis

Categorical variables were summarized as counts and percentages and age as mean and standard deviation. OS was estimated by Kaplan-Meier methods and compared using log-rank tests. Follow-up was summarized with reverse Kaplan-Meier estimates, treating censoring in the OS analysis as the event. The primary Cox model included age group, sex, race, marital status, diagnosis period, and histologic subtype (17). Treatment was omitted from the primary model because registry treatment records are incomplete, treatment selection may depend on health status, and treatment can lie on the pathway between age and survival.

Proportional hazards were assessed with scaled Schoenfeld residuals, using joint tests for multi-category variables (18). PH was not supported, so conventional HRs are treated as summary associations over follow-up rather than constant hazards; an exploratory model let the age coefficients differ between the first year and all later time. One year provides an interpretable division between early and later follow-up, but its prospective prespecification was not documented. This analysis was not intended to identify a natural change point; different cut-points could give different estimates. For counting-process models only, zero-month survival was set to 1/365.25 years to create positive time intervals. Other covariates retained constant coefficients; splitting follow-up does not by itself resolve their non-proportionality.

We modeled continuous age with a natural spline using 4 degrees of freedom and the same adjustment covariates as the primary Cox model. A likelihood-ratio comparison with linear age tested departure from linearity on the log-hazard scale. The displayed HR was referenced to age 40, an interior age rather than the lower boundary of 18. This plotting reference leaves the fitted model unchanged; the categorical reference remains 18–39 years. Pointwise confidence limits used the contrast variance (x_age-x_40)’ V (x_age-x_40), where V is the coefficient covariance matrix. The HR and both limits therefore equal 1 at age 40. The 207 records top-coded at age >=85 were represented as age 87; we also analyzed the cohort without these records.

Period-specific RMST was the unadjusted Kaplan-Meier area from diagnosis to 3 years, calculated with survival::survfit() and summary (…, rmean=3), using R version 4.4.1 and survival version 3.6-4 (14, 15). Standard errors used the integrated Greenwood covariance. For disjoint period cohorts, the newer-minus-reference difference was Delta=RMST_newer-RMST_reference, with SE_Delta=sqrt(SE_newer^2+SE_reference^2), 95% CI=Delta +/- 1.96*SE_Delta, and two-sided P = 2*Phi (-abs(Delta/SE_Delta)). We used no bootstrap, covariate adjustment, or multiplicity correction. Some patients in each diagnosis period had observed follow-up beyond 3 years, allowing estimation of a 3-year RMST; a 2-year RMST sensitivity analysis was added because the latest period had denser follow-up at 2 years. Key covariate distributions by diagnosis period were summarized with maximum pairwise standardized mean differences (SMDs). Independent censoring within periods remains necessary, and a common horizon does not adjust for changing case mix or remove uncertainty from sparse later follow-up (**Supplementary Table S3**).

We fitted Fine-Gray models for ALL/LBL-attributable and other/unknown-COD death. Covariates were age group, sex, race (White, Black, other/unknown; collapsed for parsimony), marital status (married, single, other/unknown), diagnosis period, and histologic group (B-ALL/LBL NOS, genetically defined B-ALL/LBL, T-ALL/LBL). Reference categories were 18–39 years, male, White, married, 2010-2014, and B-ALL/LBL NOS. The primary fits used the pooled censoring estimate in cmprsk::crr. We checked sensitivity to estimating censoring separately by diagnosis period. We also fitted exploratory age-by-log(1+t) terms, with t in years, and jointly tested the three terms using a Wald test to assess age-related departures from proportional subdistribution hazards (**Supplementary Table S8**). These tests address the specified log-time form and do not rule out other departures from proportionality. Because proportional subdistribution hazards were not fully supported for all endpoints, the reported SHRs should be interpreted as model-based summary associations rather than constant effects over follow-up.

Cause-specific Cox models used exactly the same three-group histology coding and other covariates as the Fine-Gray models, with Efron handling of ties. Competing deaths were censored at their observed times. Both original and unknown-COD-censored endpoint definitions were examined. We report term-specific PH diagnostics and exploratory age-by-time estimates using the same 1-year split as for OS (**Supplementary Table S7**). Censoring unknown-COD deaths is an alternative assumption, not a way to recover their true causes.

Subgroup models were fitted within sex, diagnosis-period, and histologic strata, adjusting for all primary-model covariates except the variable defining the subgroup. B-lineage models additionally adjusted for the constituent histologic subtypes. Likelihood-ratio tests evaluated age-by-period and age-by-subtype interactions. These analyses were exploratory and were not adjusted for multiple comparisons.

Sensitivity analyses restricted diagnosis to 2010-2019; excluded unknown race or marital status, other genetically defined B-ALL/LBL, or top-coded ages; re-included Burkitt; or added chemotherapy and radiation to the primary model. In the treatment-adjusted analysis, radiation was re-coded as documented administration versus none/unknown, with refusals and recommendations with uncertain administration included in none/unknown. There were 88 records with unknown race, 418 with unknown marital status, and 488 with either; their exclusion left 9,003 patients. Chemotherapy no/unknown was recorded in 1,010 patients. Radiation none/unknown comprised 7,950 patients, including 8 refusals and 22 recommendations with uncertain administration. Retaining unknown categories avoids imputing their true values but does not eliminate missing-data bias.

Analyses used R version 4.4.1 with survival, survminer, tableone, cmprsk, splines, ggplot2, dplyr, and readr. P values were two-sided; 0.05 was the nominal significance threshold. Interaction and sensitivity tests were not adjusted for multiple testing. Reporting follows STROBE recommendations (19).

## Results

### Patient characteristics

The cohort included 9,491 patients: 3,910 aged 18–39 years, 2,843 aged 40-59, 1,916 aged 60-74, and 822 aged >=75. There were 4,618 deaths, of which 3,828 were attributed to ALL/LBL and 790 to other or unknown causes. **Table 1** describes the demographic, histologic, and treatment-record distributions by age. Median follow-up, estimated by reverse Kaplan-Meier, was 66 months (95% CI 64-68) overall and 131 (129-133), 73 (71-75), and 22 (21-23) months for 2010-2014, 2015-2019, and 2020-2023, respectively (**Supplementary Table S3C**).

**Table 1:** Baseline demographic and clinical characteristics of adult patients with ALL/LBL stratified by age group.

Characteristic	Level	18-39	40-59	60-74	>=75
n		3910	2843	1916	822
Age at diagnosis, mean (SD)		26.99 (6.57)	49.81 (5.76)	66.21 (4.26)	80.79 (4.36)
Sex, n (%)	Male	2545 (65.1)	1567 (55.1)	978 (51.0)	374 (45.5)
	Female	1365 (34.9)	1276 (44.9)	938 (49.0)	448 (54.5)
Race, n (%)	American Indian/Alaska Native	72 (1.8)	36 (1.3)	24 (1.3)	4 (0.5)
	Asian or Pacific Islander	403 (10.3)	260 (9.1)	176 (9.2)	74 (9.0)
	Black	299 (7.6)	260 (9.1)	147 (7.7)	39 (4.7)
	Unknown	50 (1.3)	21 (0.7)	11 (0.6)	6 (0.7)
	White	3086 (78.9)	2266 (79.7)	1558 (81.3)	699 (85.0)
Marital status, n (%)	Married	1109 (28.4)	1714 (60.3)	1173 (61.2)	396 (48.2)
	Single	2566 (65.6)	674 (23.7)	285 (14.9)	78 (9.5)
	Divorced/Sep/Widowed	101 (2.6)	327 (11.5)	359 (18.7)	291 (35.4)
	Unknown	134 (3.4)	128 (4.5)	99 (5.2)	57 (6.9)
Diagnosis period, n (%)	2010-2014	1253 (32.0)	972 (34.2)	575 (30.0)	277 (33.7)
	2015-2019	1469 (37.6)	987 (34.7)	685 (35.8)	298 (36.3)
	2020-2023	1188 (30.4)	884 (31.1)	656 (34.2)	247 (30.0)
Histologic subtype, n (%)	B-ALL/LBL with BCR-ABL1	247 (6.3)	366 (12.9)	255 (13.3)	65 (7.9)
	B-ALL/LBL, NOS	2556 (65.4)	1958 (68.9)	1374 (71.7)	626 (76.2)
	B-ALL/LBL, other genetic subtypes	163 (4.2)	109 (3.8)	76 (4.0)	32 (3.9)
	T-ALL/LBL	944 (24.1)	410 (14.4)	211 (11.0)	99 (12.0)
Chemotherapy recorded, n (%)	No or unknown	150 (3.8)	176 (6.2)	299 (15.6)	385 (46.8)
	Yes	3760 (96.2)	2667 (93.8)	1617 (84.4)	437 (53.2)
Radiation recorded, n (%)	None or unknown	3077 (78.7)	2269 (79.8)	1792 (93.5)	812 (98.8)
	Yes	833 (21.3)	574 (20.2)	124 (6.5)	10 (1.2)

Values are n (%) unless stated otherwise. Ages >=85 were represented as 87 when calculating mean age and SD. Radiation none/unknown includes refusal (n=8) and recommendation with uncertain administration (n=22). Percentages may not total 100% because of rounding. NOS, not otherwise specified; SD, standard deviation.

### Overall survival analysis

OS was lower in older age groups (**Figure 1**). The three diagnosis periods included 3,077, 3,439, and 2,975 patients, with 1,958, 1,732, and 928 deaths, respectively. Median OS was 30 months in 2010-2014, 65 months in 2015-2019, and not reached in 2020-2023. Corresponding 1-year OS was 65.7%, 73.7%, and 74.0%; 3-year OS was 47.3%, 55.9%, and 58.3% (**Figure 2**).

**Figure 1:**
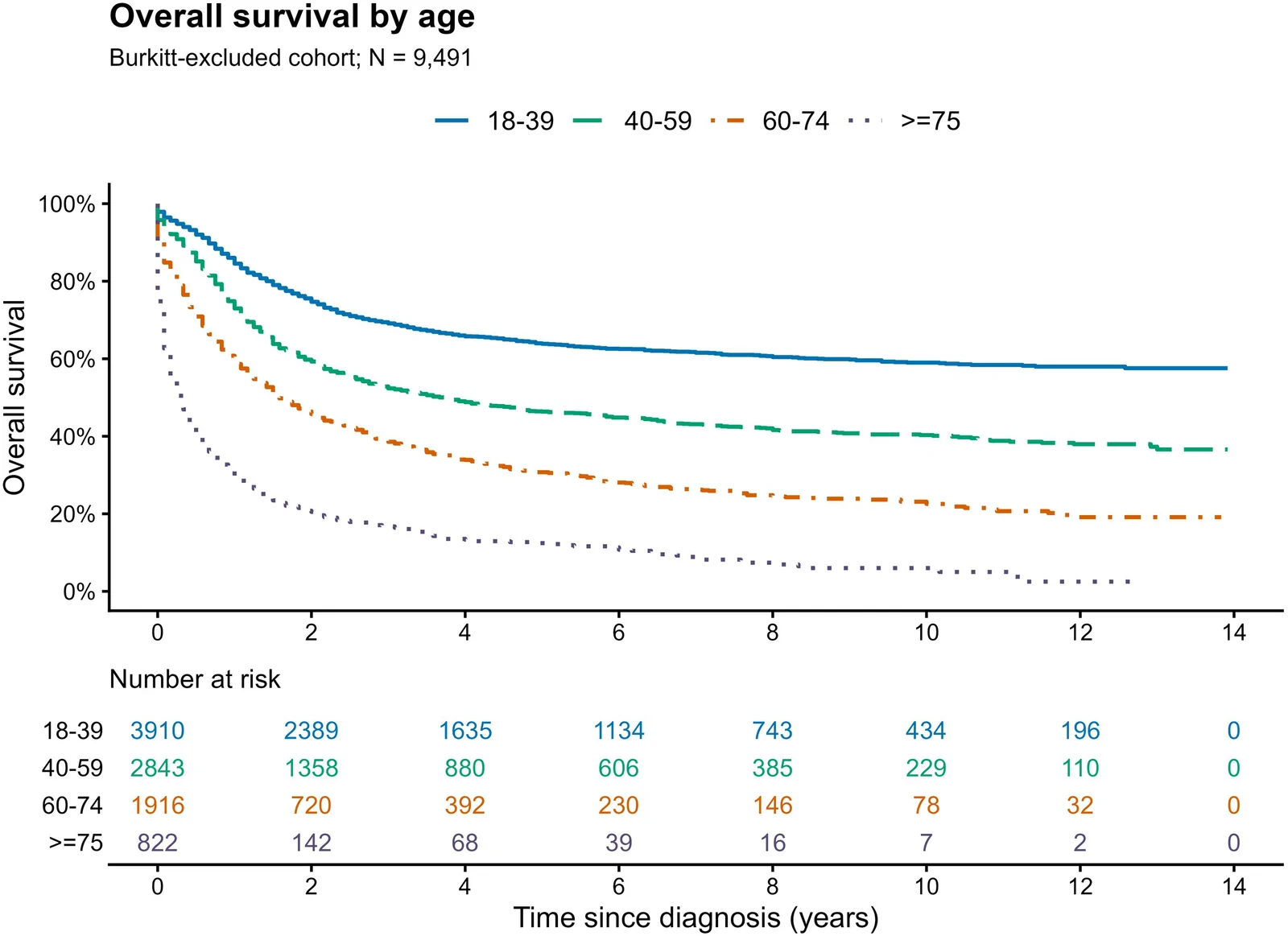
Kaplan-Meier overall survival by age group. The final cohort comprises 9,491 adults with ALL/LBL after exclusion of Burkitt (9826). Numbers at risk are shown below the curves; curves stop at the available follow-up. Line patterns and colors identify age groups. These unadjusted curves do not assume proportional hazards.

**Figure 2:**
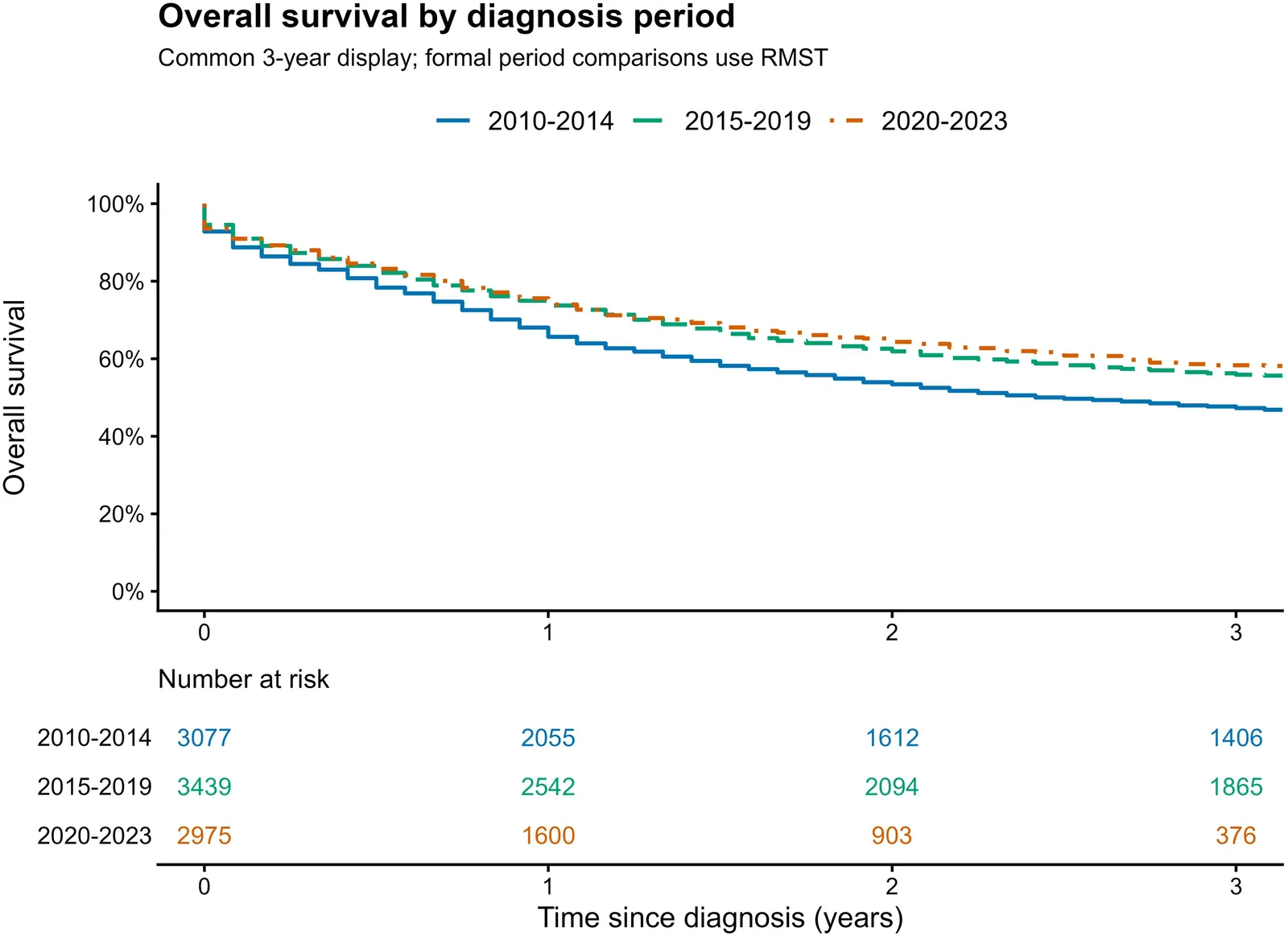
Kaplan-Meier overall survival by diagnosis period, displayed through a common 3-year horizon. Numbers at risk are shown below the curves. The formal period comparison uses unadjusted 3-year RMST (**Supplementary Table S3**). The 2020–2023 versus 2015–2019 RMST difference is 0.044 years (95% CI -0.016 to 0.104; P = 0.150). The recent-period estimate relies on independent censoring and 376 patients at risk at 3 years.

Three-year RMST was 1.89, 2.10, and 2.14 years, respectively. Compared with 2010-2014, the difference was 0.21 years for 2015-2019 (95% CI 0.15-0.26; P = 2.01e-12) and 0.25 years for 2020-2023 (95% CI 0.19-0.31; P = 3.43e-15). The 2020–2023 versus 2015–2019 difference was 0.044 years (95% CI -0.016 to 0.104; P = 0.150). At 3 years, 1,406, 1,865, and 376 patients remained at risk. A 2-year RMST sensitivity analysis showed the same qualitative pattern; the 2020–2023 versus 2015–2019 difference was 0.019 years (95% CI -0.018 to 0.055; P = 0.322; **Supplementary Tables S3D, E**). **Figure 2** is limited to the common 3-year horizon; period-specific estimates, contrasts, follow-up support, and case-mix distributions are in **Supplementary Tables S3A–F**.

### Cox regression analysis

Compared with age 18–39 years, adjusted HRs for all-cause mortality were 1.792 (95% CI 1.648-1.949), 2.910 (2.661-3.182), and 6.258 (5.627-6.960) for ages 40-59, 60-74, and >=75, respectively (all P<0.001; **Table 2**). Because PH was not supported, these estimates summarize associations over follow-up.

**Table 2:** Primary multivariable Cox proportional hazards model for overall survival in adult ALL/LBL.

Covariate	HR	95% CI lower	95% CI upper	P value
Age 40–59 years	1.792	1.648	1.949	2.36e-42
Age 60–74 years	2.910	2.661	3.182	3.09e-121
Age >=75 years	6.258	5.627	6.960	1.13e-250
Female sex	0.888	0.836	0.942	8.80e-05
Race: American Indian/Alaska Native	1.116	0.874	1.425	0.378
Race: Asian or Pacific Islander	0.835	0.751	0.928	8.58e-4
Race: Black	1.226	1.105	1.361	1.22e-4
Race: Unknown	0.492	0.309	0.782	0.00273
Marital status: Single	1.097	1.017	1.183	0.0166
Marital status: Divorced/separated/widowed	1.301	1.194	1.418	1.95e-9
Marital status: Unknown	1.020	0.886	1.175	0.781
Diagnosis period: 2015-2019	0.779	0.730	0.832	9.11e-14
Diagnosis period: 2020-2023	0.711	0.656	0.771	1.14e-16
Subtype: B-ALL/LBL with BCR-ABL1	0.751	0.676	0.835	1.08e-7
Subtype: B-ALL/LBL, other genetic subtypes	0.863	0.731	1.018	0.0805
Subtype: T-ALL/LBL	0.866	0.796	0.941	6.97e-4

All covariates shown were entered together; treatment was not included. References: age 18–39 years, male, White, married, diagnosis in 2010-2014, and B-ALL/LBL NOS. HRs are summary associations because PH was violated (**Supplementary Tables S1**, **S5**). HR, hazard ratio; CI, confidence interval.

In descriptive conventional Cox analyses, diagnosis in 2015–2019 and 2020–2023 was associated with lower overall mortality than diagnosis in 2010-2014 (HR 0.779, 95% CI 0.730-0.832, and HR 0.711, 95% CI 0.656-0.771, respectively). Female sex was also associated with lower mortality (HR 0.888, 95% CI 0.836-0.942). All three P values were <0.001. Because proportional hazards and equal follow-up were not assured, these estimates were not used as the primary comparison of temporal survival differences; the common-horizon RMST analysis was treated as the primary period comparison.

The global Schoenfeld test rejected PH (chi-square=134.264, df=16, P = 9.51e-21). Term-specific P values were 1.22e-21 for age (chi-square=100.495, df=3), 0.0269 for sex, 0.3917 for race, 2.58e-05 for marital status, 0.0479 for period, and 0.0035 for histologic subtype (**Supplementary Table S1**). In the exploratory time-split model, the >=75-year HR was 8.201 (95% CI 7.224-9.311) during 0–1 year and 3.496 (2.885-4.236) after 1 year; the joint age-by-time likelihood-ratio P value was 1.45e-15 (**Supplementary Table S5**). Thus, the conventional HR of 6.258 does not describe a constant relative hazard. Neither the nonsignificant race test nor a single time split establishes proportionality.

### Competing-risk analysis

Compared with age 18–39 years, ALL/LBL-attributable SHRs were 1.659 (95% CI 1.517-1.815), 2.386 (2.164-2.631), and 4.518 (3.986-5.121) for ages 40-59, 60-74, and >=75, respectively. Corresponding other/unknown-COD SHRs were 1.778 (1.429-2.211), 3.054 (2.447-3.811), and 3.470 (2.673-4.504), all P<0.001 (**Table 3**). These estimates concern subdistribution hazards, not individual probabilities of death.

**Table 3:** Fine-Gray models for ALL/LBL-attributable and other/unknown-cause death, with an unknown-COD sensitivity analysis.

Covariate	SHR	95% CI lower	95% CI upper	P value	Outcome	Cause-of-death handling
Age 40–59 years	1.659	1.517	1.815	1.69e-28	ALL/LBL-attributable death	A: unknown COD as other
Age 60–74 years	2.386	2.164	2.631	3.75e-68	ALL/LBL-attributable death	A: unknown COD as other
Age >=75 years	4.518	3.986	5.121	4.41e-123	ALL/LBL-attributable death	A: unknown COD as other
Female sex	0.946	0.885	1.010	0.0974	ALL/LBL-attributable death	A: unknown COD as other
Race: Black	1.095	0.973	1.233	0.132	ALL/LBL-attributable death	A: unknown COD as other
Race: Other/unknown	0.826	0.741	0.920	5.43e-4	ALL/LBL-attributable death	A: unknown COD as other
Marital status: Single	1.071	0.986	1.163	0.106	ALL/LBL-attributable death	A: unknown COD as other
Marital status: Other/unknown	1.180	1.078	1.292	3.29e-4	ALL/LBL-attributable death	A: unknown COD as other
Diagnosis period: 2015-2019	0.758	0.705	0.815	5.80e-14	ALL/LBL-attributable death	A: unknown COD as other
Diagnosis period: 2020-2023	0.639	0.585	0.699	9.22e-23	ALL/LBL-attributable death	A: unknown COD as other
Subtype: B-ALL/LBL, genetically defined	0.802	0.725	0.886	1.43e-5	ALL/LBL-attributable death	A: unknown COD as other
Subtype: T-ALL/LBL	0.907	0.828	0.994	0.0367	ALL/LBL-attributable death	A: unknown COD as other
Age 40–59 years	1.778	1.429	2.211	2.35e-7	Other/unknown-COD death	A: unknown COD as other
Age 60–74 years	3.054	2.447	3.811	5.06e-23	Other/unknown-COD death	A: unknown COD as other
Age >=75 years	3.470	2.673	4.504	8.74e-21	Other/unknown-COD death	A: unknown COD as other
Female sex	0.794	0.686	0.917	0.00179	Other/unknown-COD death	A: unknown COD as other
Race: Black	1.539	1.218	1.945	3.01e-4	Other/unknown-COD death	A: unknown COD as other
Race: Other/unknown	1.040	0.833	1.299	0.729	Other/unknown-COD death	A: unknown COD as other
Marital status: Single	1.083	0.895	1.310	0.414	Other/unknown-COD death	A: unknown COD as other
Marital status: Other/unknown	1.189	0.986	1.434	0.0701	Other/unknown-COD death	A: unknown COD as other
Diagnosis period: 2015-2019	0.899	0.769	1.050	0.179	Other/unknown-COD death	A: unknown COD as other
Diagnosis period: 2020-2023	0.831	0.690	1.001	0.0517	Other/unknown-COD death	A: unknown COD as other
Subtype: B-ALL/LBL, genetically defined	0.906	0.733	1.121	0.364	Other/unknown-COD death	A: unknown COD as other
Subtype: T-ALL/LBL	0.752	0.610	0.928	0.00788	Other/unknown-COD death	A: unknown COD as other
Age 40–59 years	1.662	1.519	1.818	1.31e-28	ALL/LBL-attributable death	B: unknown COD censored
Age 60–74 years	2.406	2.182	2.653	2.57e-69	ALL/LBL-attributable death	B: unknown COD censored
Age >=75 years	4.567	4.030	5.177	6.21e-125	ALL/LBL-attributable death	B: unknown COD censored
Female sex	0.943	0.883	1.008	0.0842	ALL/LBL-attributable death	B: unknown COD censored
Race: Black	1.097	0.975	1.235	0.125	ALL/LBL-attributable death	B: unknown COD censored
Race: Other/unknown	0.826	0.741	0.920	5.46e-4	ALL/LBL-attributable death	B: unknown COD censored
Marital status: Single	1.074	0.989	1.167	0.0896	ALL/LBL-attributable death	B: unknown COD censored
Marital status: Other/unknown	1.182	1.080	1.294	2.84e-4	ALL/LBL-attributable death	B: unknown COD censored
Diagnosis period: 2015-2019	0.760	0.707	0.816	8.79e-14	ALL/LBL-attributable death	B: unknown COD censored
Diagnosis period: 2020-2023	0.640	0.586	0.700	1.51e-22	ALL/LBL-attributable death	B: unknown COD censored
Subtype: B-ALL/LBL, genetically defined	0.799	0.723	0.883	1.06e-5	ALL/LBL-attributable death	B: unknown COD censored
Subtype: T-ALL/LBL	0.906	0.827	0.993	0.0343	ALL/LBL-attributable death	B: unknown COD censored
Age 40–59 years	1.938	1.525	2.464	6.55e-8	Known other-cause death	B: unknown COD censored
Age 60–74 years	3.421	2.686	4.358	2.28e-23	Known other-cause death	B: unknown COD censored
Age >=75 years	4.014	3.031	5.315	3.12e-22	Known other-cause death	B: unknown COD censored
Female sex	0.808	0.692	0.945	0.00755	Known other-cause death	B: unknown COD censored
Race: Black	1.633	1.277	2.089	9.38e-5	Known other-cause death	B: unknown COD censored
Race: Other/unknown	1.003	0.785	1.281	0.981	Known other-cause death	B: unknown COD censored
Marital status: Single	1.086	0.884	1.333	0.432	Known other-cause death	B: unknown COD censored
Marital status: Other/unknown	1.193	0.977	1.457	0.0840	Known other-cause death	B: unknown COD censored
Diagnosis period: 2015-2019	0.879	0.744	1.040	0.133	Known other-cause death	B: unknown COD censored
Diagnosis period: 2020-2023	0.853	0.700	1.040	0.115	Known other-cause death	B: unknown COD censored
Subtype: B-ALL/LBL, genetically defined	0.943	0.754	1.179	0.606	Known other-cause death	B: unknown COD censored
Subtype: T-ALL/LBL	0.693	0.548	0.876	0.00220	Known other-cause death	B: unknown COD censored

References: age 18–39 years, male, White, married, 2010-2014, and B-ALL/LBL NOS. Genetically defined B-ALL/LBL combines code 9812 and codes 9813-9819; the third group is T-ALL/LBL. Coding A classifies 106 unknown-COD deaths with other-cause deaths; coding B censors them at death. SHRs are not risk ratios or cause-specific HRs. Because proportional subdistribution hazards were not fully supported for all endpoints, SHRs should be interpreted as model-based summary associations rather than constant effects over follow-up. Age-related proportionality and period-specific censoring checks are in **Supplementary Table S8**. SHR, subdistribution hazard ratio; CI, confidence interval.

The ALL/LBL-attributable SHRs for 2015–2019 and 2020–2023 versus 2010–2014 were 0.758 (95% CI 0.705-0.815) and 0.639 (0.585-0.699). These period SHRs share the unequal follow-up windows noted for the Cox model and are descriptive. Censoring the 106 unknown-COD deaths gave >=75-year SHRs of 4.567 (4.030-5.177) for ALL/LBL-attributable death and 4.014 (3.031-5.315) for known other-cause death (**Table 3**).

The exploratory age-by-log(1+t) test detected departure from proportional subdistribution hazards for ALL/LBL-attributable death (Wald chi-square=170.223, df=3, P = 1.14e-36), but not for other/unknown-COD death (chi-square=4.888, df=3, P = 0.180). The latter result should not be taken as proof of proportionality. Because proportional subdistribution hazards were not fully supported for all endpoints, the reported SHRs are model-based summary associations rather than constant effects over follow-up. With censoring estimated separately by diagnosis period, the >=75-year SHRs were 4.468 (95% CI 3.938-5.070) and 3.377 (2.601-4.384), respectively (**Supplementary Table S8**). We therefore emphasize fixed-time cumulative incidence when describing absolute mortality.

In cause-specific Cox models using the same adjustment categories, >=75-year summary HRs were 5.833 (95% CI 5.197-6.547) for ALL/LBL-attributable death and 9.032 (6.935-11.764) for other/unknown-COD death. Censoring unknown-COD deaths left the ALL/LBL-attributable estimate unchanged and gave HR 10.340 (7.776-13.750) for known other-cause death, based on 684 events (**Supplementary Table S7A**). The ALL/LBL-attributable estimate is unchanged because these deaths already leave its cause-specific risk set at death.

Age violated cause-specific PH for ALL/LBL-attributable death (P = 4.69e-35) and other/unknown-COD death (P = 0.0106; **Supplementary Table S7B**). The >=75-year ALL/LBL-attributable HR was 8.323 (95% CI 7.264-9.537) during the first year and 2.406 (1.897-3.052) thereafter. Corresponding other/unknown-COD HRs were 7.190 (5.103-10.130) and 11.327 (7.905-16.231). This opposite time pattern is compatible with ALL/LBL-attributable death dominating the first year, leaving competing causes a larger share among first-year survivors. Joint age-by-time P values were 9.72e-25 and 0.196, respectively (**Supplementary Tables S7C, D**). A nonsignificant interaction at this cut-point does not re-establish proportional hazards.

### Cumulative incidence analysis

At 1, 3, and 5 years, ALL/LBL-attributable cumulative incidence was 13.3%, 26.7%, and 31.3% in patients aged 18–39 years, compared with 61.6%, 70.4%, and 72.2% in those aged >=75. Corresponding other/unknown-COD estimates were 2.2%, 4.2%, and 4.8% versus 8.8%, 12.8%, and 15.3% (**Figure 3**). These are fixed-time event probabilities accounting for competing death.

**Figure 3:**
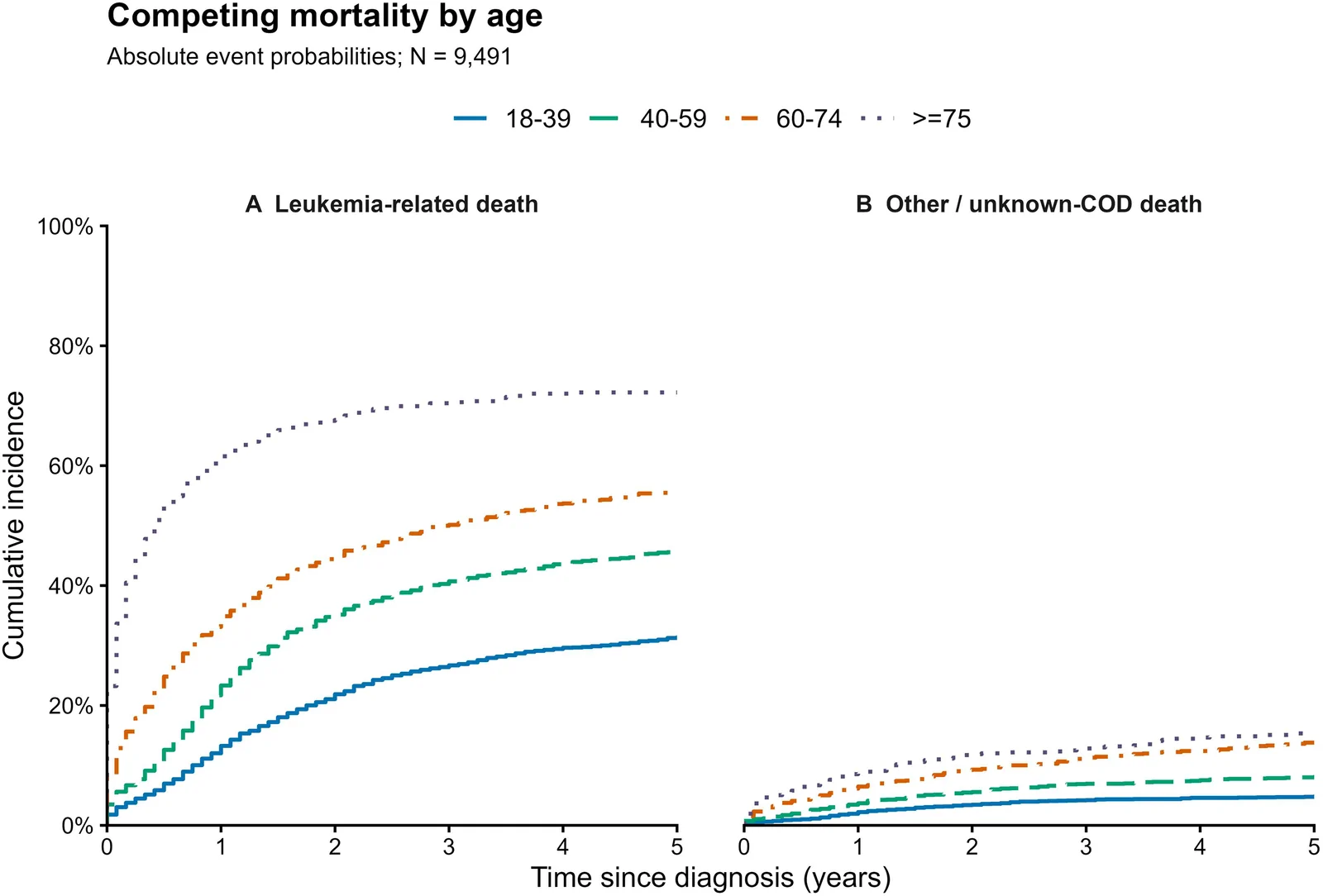
Cumulative incidence through 5 years by age: **(A)** ALL/LBL-attributable death and **(B)** other/unknown-COD death. Curves account for competing death and show absolute event probabilities. The primary other/unknown-COD endpoint includes 106 deaths of unknown cause; **Table 3** and **Supplementary Table S7** report sensitivity analyses censoring these deaths. Colors and line patterns identify age groups.

### Nonlinear association between age and overall mortality

The spline model supported departure from a linear age term on the log-hazard scale (likelihood-ratio chi-square=60.54, df=3, P = 4.51e-13). The fitted summary HR increased with age (**Figure 4**). This comparison establishes neither a biological age threshold nor a constant age association over follow-up.

**Figure 4:**
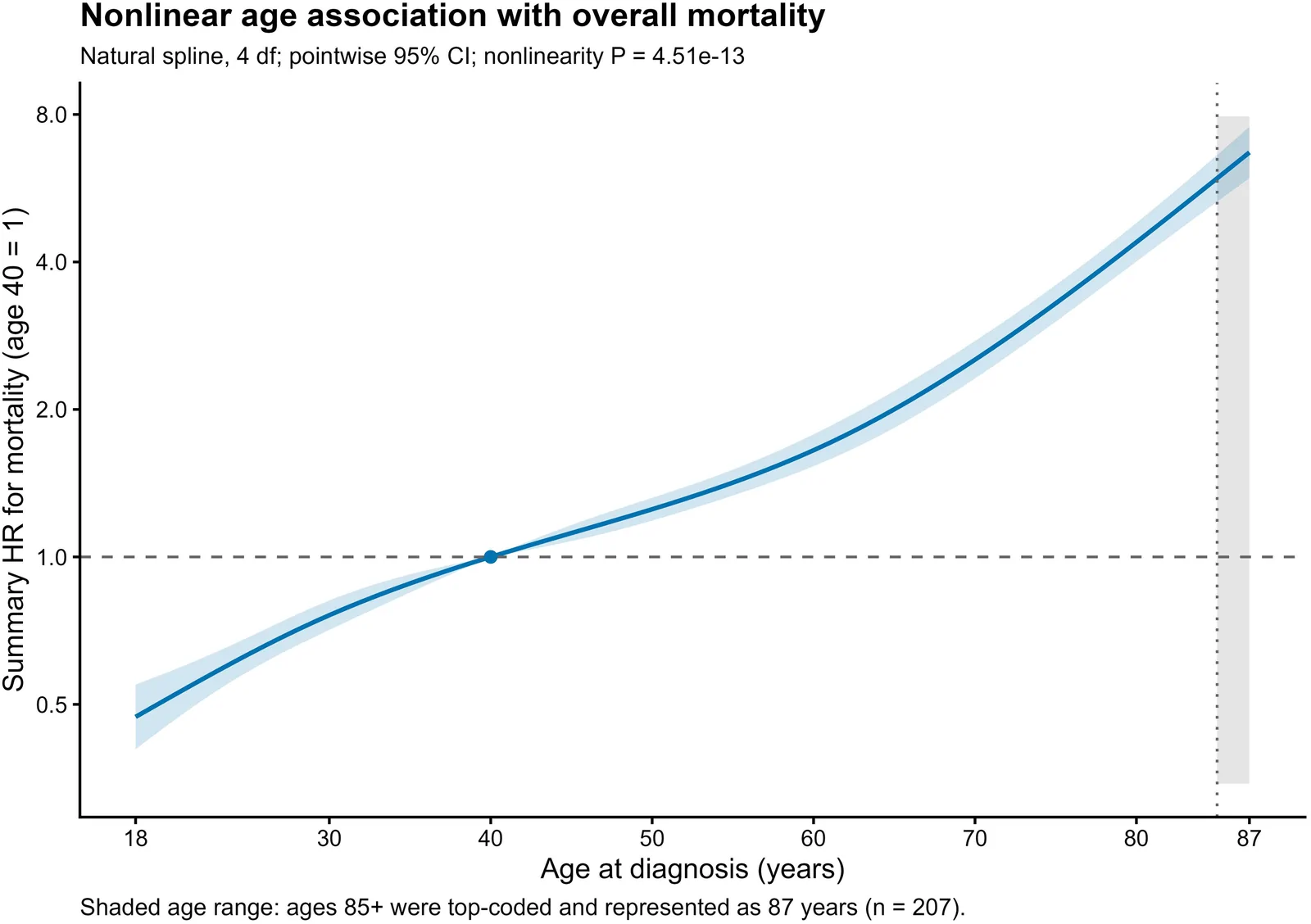
Natural-spline association of age with overall mortality. The model uses 4 spline degrees of freedom and adjusts for sex, race, marital status, diagnosis period, and histologic subtype. Age 40 is the reference; the ribbon is the pointwise 95% CI from the age-versus-40 contrast covariance. HR and CI limits equal 1 at the reference. The likelihood-ratio comparison with linear age tests departure from linearity on the log-hazard scale (chi-square=60.54, df=3, P = 4.51e-13). Shading identifies top-coded ages (207 patients aged >=85 represented as 87). HRs are follow-up summary associations because PH was violated.

### Cause-of-death composition

At last follow-up, the proportions alive, dead from ALL/LBL, and dead from other or unknown causes were 67.3%, 28.1%, and 4.7% at ages 18-39; 50.9%, 41.0%, and 8.1% at ages 40-59; 35.2%, 51.5%, and 13.3% at ages 60-74; and 14.8%, 70.2%, and 15.0% at ages >=75 (**Figure 5**). Denominators are all patients in each age group, not only those who died. These crude proportions reflect each group’s observed follow-up and are distinct from cumulative incidence at 3 or 5 years.

**Figure 5:**
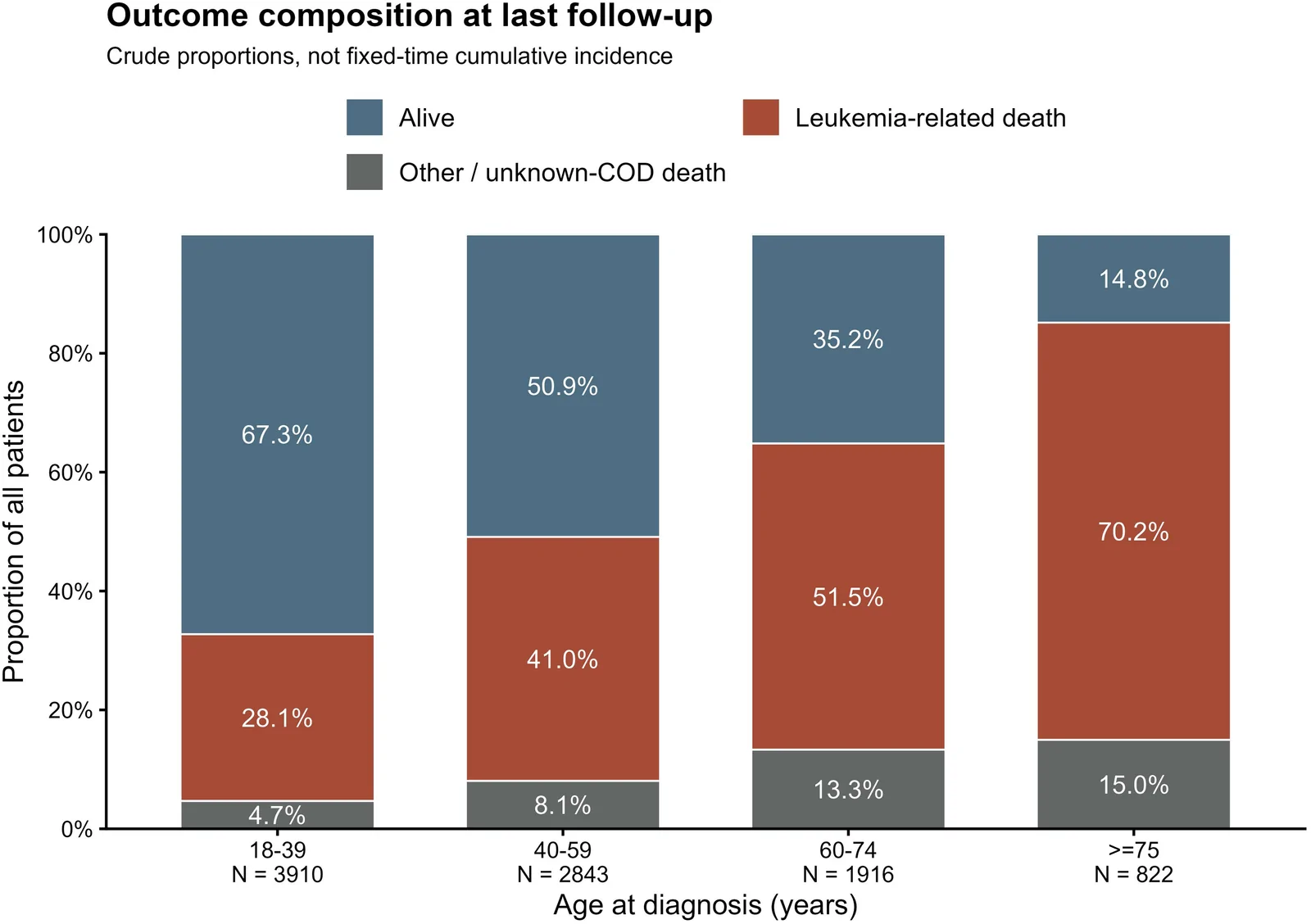
Outcome composition at last follow-up. Percentages use all patients in each age group as the denominator and are crude observed proportions, not fixed-time cumulative incidence. The other/unknown-COD category includes deaths of unknown cause. Among patients aged >=75, 577/822 (70.2%) had ALL/LBL-attributable death, 123/822 (15.0%) had other/unknown-COD death, and 122/822 (14.8%) were alive. Percentages may not sum to 100% because of rounding.

### Subgroup analysis

Older age was associated with higher mortality within each sex, period, and histologic subgroup (**Table 4**). For age >=75 versus 18–39 years, HRs were 6.715 (95% CI 5.798-7.776) among males and 5.643 (4.823-6.604) among females. These separate estimates do not by themselves test an age-by-sex interaction.

**Table 4:** Subgroup and sensitivity analyses of the association between age group and overall survival.

Section	Subgroup or analysis	Age comparison	HR	95% CI lower	95% CI upper	P value	N (cohort size)
Subgroup	Sex: Male	40-59	1.893	1.695	2.114	1.26e-29	5464
Subgroup	Sex: Male	60-74	3.172	2.816	3.574	2.05e-80	5464
Subgroup	Sex: Male	>=75	6.715	5.798	7.776	1.06e-142	5464
Subgroup	Sex: Female	40-59	1.645	1.445	1.873	5.19e-14	4027
Subgroup	Sex: Female	60-74	2.572	2.245	2.947	3.69e-42	4027
Subgroup	Sex: Female	>=75	5.643	4.823	6.604	3.04e-103	4027
Subgroup	Period: 2010-2014	40-59	1.801	1.589	2.041	3.31e-20	3077
Subgroup	Period: 2010-2014	60-74	2.690	2.341	3.091	2.87e-44	3077
Subgroup	Period: 2010-2014	>=75	5.942	5.024	7.028	3.11e-96	3077
Subgroup	Period: 2015-2019	40-59	1.682	1.464	1.933	2.17e-13	3439
Subgroup	Period: 2015-2019	60-74	3.044	2.631	3.520	8.78e-51	3439
Subgroup	Period: 2015-2019	>=75	6.681	5.618	7.945	2.21e-102	3439
Subgroup	Period: 2020-2023	40-59	2.017	1.657	2.455	2.75e-12	2975
Subgroup	Period: 2020-2023	60-74	3.238	2.652	3.954	8.65e-31	2975
Subgroup	Period: 2020-2023	>=75	6.547	5.189	8.260	1.53e-56	2975
Subgroup	Subtype: B-ALL/LBL with BCR-ABL1	40-59	1.797	1.313	2.459	2.52e-4	933
Subgroup	Subtype: B-ALL/LBL with BCR-ABL1	60-74	2.640	1.915	3.641	3.20e-9	933
Subgroup	Subtype: B-ALL/LBL with BCR-ABL1	>=75	7.037	4.651	10.647	2.62e-20	933
Subgroup	Subtype: B-ALL/LBL, NOS	40-59	1.692	1.534	1.866	7.51e-26	6514
Subgroup	Subtype: B-ALL/LBL, NOS	60-74	2.792	2.518	3.097	3.62e-84	6514
Subgroup	Subtype: B-ALL/LBL, NOS	>=75	5.645	4.994	6.380	1.21e-168	6514
Subgroup	Subtype: B-ALL/LBL, other genetic subtypes	40-59	1.974	1.180	3.302	0.00957	380
Subgroup	Subtype: B-ALL/LBL, other genetic subtypes	60-74	3.217	1.918	5.396	9.45e-6	380
Subgroup	Subtype: B-ALL/LBL, other genetic subtypes	>=75	7.749	4.065	14.774	4.98e-10	380
Subgroup	Subtype: T-ALL/LBL	40-59	2.135	1.736	2.625	6.36e-13	1664
Subgroup	Subtype: T-ALL/LBL	60-74	3.553	2.796	4.516	3.47e-25	1664
Subgroup	Subtype: T-ALL/LBL	>=75	10.221	7.772	13.440	3.78e-62	1664
Sensitivity	Main cohort (no treatment adjustment)	40-59	1.792	1.648	1.949	2.36e-42	9491
Sensitivity	Main cohort (no treatment adjustment)	60-74	2.910	2.661	3.182	3.09e-121	9491
Sensitivity	Main cohort (no treatment adjustment)	>=75	6.258	5.627	6.960	1.13e-250	9491
Sensitivity	Restrict to 2010-2019	40-59	1.748	1.593	1.918	4.63e-32	6516
Sensitivity	Restrict to 2010-2019	60-74	2.854	2.582	3.155	3.10e-93	6516
Sensitivity	Restrict to 2010-2019	>=75	6.247	5.540	7.045	3.41e-196	6516
Sensitivity	Exclude unknown race/marital status	40-59	1.792	1.645	1.953	2.65e-40	9003
Sensitivity	Exclude unknown race/marital status	60-74	2.941	2.683	3.224	2.90e-117	9003
Sensitivity	Exclude unknown race/marital status	>=75	6.404	5.737	7.148	3.93e-240	9003
Sensitivity	Exclude rare histologic subtypes (other-genetic only)	40-59	1.783	1.638	1.942	1.62e-40	9111
Sensitivity	Exclude rare histologic subtypes (other-genetic only)	60-74	2.902	2.650	3.179	9.13e-117	9111
Sensitivity	Exclude rare histologic subtypes (other-genetic only)	>=75	6.240	5.600	6.952	9.10e-242	9111
Sensitivity	Re-include Burkitt (broader code set)	40-59	1.802	1.660	1.957	1.43e-44	9743
Sensitivity	Re-include Burkitt (broader code set)	60-74	2.877	2.634	3.143	9.00e-122	9743
Sensitivity	Re-include Burkitt (broader code set)	>=75	6.394	5.761	7.096	6.90e-267	9743
Sensitivity	Exclude 85+ top-coded	40-59	1.800	1.655	1.958	8.59e-43	9284
Sensitivity	Exclude 85+ top-coded	60-74	2.932	2.680	3.207	5.70e-122	9284
Sensitivity	Exclude 85+ top-coded	>=75	5.660	5.042	6.353	6.32e-190	9284
Sensitivity	Treatment-adjusted (chemo + radio)	40-59	1.738	1.598	1.889	3.67e-38	9491
Sensitivity	Treatment-adjusted (chemo + radio)	60-74	2.499	2.282	2.737	1.22e-86	9491
Sensitivity	Treatment-adjusted (chemo + radio)	>=75	4.473	3.988	5.017	2.04e-144	9491

Age 18–39 years is the reference in every model. Subgroup models adjust for all primary-model covariates except the variable defining the subgroup; sex is retained in period and histologic models. The treatment-adjusted analysis adds chemotherapy and radiation recoded as documented administration versus none/unknown, including refusals and recommendations with uncertain administration. HRs summarize associations under non-proportional hazards. The T-ALL/LBL estimate equals the T-lineage estimate in **Supplementary Table S6**.

The age-by-period interaction was not significant (likelihood-ratio P = 0.182); the exploratory age-by-subtype interaction was nominally significant (P = 0.024). The >=75-year HRs were 5.645 (95% CI 4.994-6.380) for B-ALL/LBL NOS, 7.037 (4.651-10.647) for B-ALL/LBL with BCR-ABL1, 7.749 (4.065-14.774) for other genetically defined B-ALL/LBL, and 10.221 (7.772-13.440) for T-ALL/LBL. The B-lineage estimate, combining the three B-lineage histologic groups, was 5.778 (5.150-6.482; n=7,827). The T-lineage analysis included the same 1,664 patients and covariates as the T-ALL/LBL subgroup and yielded the same estimate (**Supplementary Table S6**). Only 99 T-lineage patients were aged >=75; the interaction test concerns all four histologic categories and is not a dedicated B-versus-T interaction test.

### Sensitivity analysis

Across analyses that restricted the cohort or re-included Burkitt, >=75-year HRs ranged from 5.660 to 6.404 (**Table 4**; **Supplementary Table S4**). Re-including Burkitt gave HR 6.394. Adding chemotherapy and recoded radiation categories attenuated the >=75-year HR to 4.473 (95% CI 3.988-5.017). The age association remained positive but was attenuated. These treatment records do not identify the respective contributions of treatment selection, treatment-mediated pathways, and residual confounding.

## Discussion

In this ALL/LBL cohort, older age was associated with higher mortality, but a single HR did not capture how strongly that association changed over time. Among patients aged >=75, the adjusted all-cause HR was 8.201 during the first year and 3.496 thereafter. ALL/LBL-attributable death remained the more frequent endpoint at 3 years, with cumulative incidence of 70.4% versus 12.8% for other/unknown-COD death. Three-year RMST was higher in both later periods than in 2010-2014, whereas the difference between 2015–2019 and 2020–2023 remained imprecise. Taken together, the findings sharpen the description of known age disparities without establishing a new biological mechanism or validating an age-based treatment rule. The study should be viewed as a population-level prognostic and methodological analysis, not as a treatment-selection model.

RMST provides a direct comparison of survival time within a stated horizon and is useful when hazards are not proportional (14, 15). In this cohort, the recent-period difference was 0.044 years, about two weeks, with a confidence interval spanning from a small decrease to a gain of about five weeks. This is not evidence of equivalence or of a plateau in treatment progress. The 2020–2023 cohort had a median follow-up of 22 months and only 376 patients at risk at 3 years. A 2-year sensitivity analysis, supported by 903 patients at risk in 2020-2023, likewise did not establish a difference between 2015–2019 and 2020-2023. Unadjusted RMST cannot separate changes in disease mix from changes in care. Case-mix summaries (**Supplementary Table S3F**) showed that the largest period shifts involved histologic coding categories, including B-ALL/LBL with BCR-ABL1 (6.1% in 2010–2014 to 13.3% in 2020-2023; maximum SMD 0.244) and other genetically defined B-lineage subtypes (1.7% to 6.4%; maximum SMD 0.242), consistent with changing ascertainment and coding over time. The 2020–2023 period also overlapped the COVID-19 pandemic, which may have affected diagnostic timing, treatment delivery, follow-up, and other-cause mortality coding; if pandemic-related disruption worsened outcomes in the latest cohort, the null recent-period contrast may underestimate rather than overstate improvement. The more favorable comparison with 2010–2014 is consistent with published temporal trends, including a Danish study using age-standardized survival estimates (9), but the designs and follow-up windows differ.

Treatment advances help frame the calendar-period findings, but they cannot be identified as their explanation in these data. Pediatric-inspired regimens have been evaluated in adolescents and younger adults, although their adoption in adult treatment centers has varied (20–22). Studies of dasatinib-blinatumomab and ponatinib-hyper-CVAD address frontline Ph-positive ALL (23, 24); randomized blinatumomab and inotuzumab trials address relapsed or refractory B-ALL (25, 26). CAR T-cell studies include distinct adult and pediatric/young-adult populations (27, 28). Our export does not record these regimens or exposures, and findings from these settings cannot be assigned to a calendar-period coefficient or generalized directly to patients aged >=75.

The baseline differences in **Table 1** also have clinical implications. The shift in marital status with age may reflect differences in social support, treatment access, and the ability to complete prolonged therapy, although marital status is only a crude registry proxy. Histologic composition also varied by age: the oldest group had a larger proportion of B-ALL/LBL NOS and a smaller proportion of T-ALL/LBL than younger adults. Because NOS coding does not define molecular risk, these distributions should be read as registry case-mix differences rather than as complete biologic classification. The steep age gradient in chemotherapy and radiation records is clinically plausible and may reflect fitness, treatment selection, physician recommendation, patient preference, and documentation limits; it should not be interpreted as a direct treatment effect.

In the competing-risk analysis, most deaths in the oldest group were still attributed to the index ALL/LBL diagnosis. At 3 years, ALL/LBL-attributable cumulative incidence in the oldest group was more than five times the other/unknown-COD estimate. Comorbidity and treatment toxicity may still have contributed, because registry attribution cannot reliably separate these mechanisms. The crude 70.2% ALL/LBL-attributable proportion at last follow-up uses a different time basis and should not replace the 3-year estimate. Fine-Gray and cause-specific models answer different questions (12, 13); both showed time variation in the age association for ALL/LBL-attributable death. Their relative-hazard estimates are therefore best read alongside the cumulative incidence curves.

The spline analysis found that a linear age term did not adequately describe the log-hazard association. These results argue against reducing age to a purely linear term. They do not, however, show that age measures frailty or identify a treatment threshold. The >=75-year summary HR was numerically larger in T-lineage than B-lineage disease (10.221 versus 5.778), consistent with heterogeneity across coded subtypes. B- and T-lineage ALL differ biologically and therapeutically (3, 29), yet the present analysis cannot attribute the difference to those mechanisms. The small older T-lineage subgroup, different within-lineage reference populations, and time-varying hazards limit direct comparison of the HR magnitudes.

Cohort restrictions preserved the direction of the age association. Treatment adjustment attenuated it, but this cannot quantify how much of the association is mediated by treatment. Chemotherapy no/unknown and radiation none/unknown combine clinically different situations, and treatment timing, intensity, and fitness for treatment were unavailable. SEER chemotherapy coding also does not reliably capture oral targeted agents. The exploratory time-split results describe where the relative association was larger; they do not establish a safe or biologically distinct phase after 1 year.

The subgroup and sensitivity analyses mainly serve as boundary checks. Higher mortality persisted across sex, diagnosis-period, and histologic strata, suggesting that age remains relevant even after broad demographic and disease-code differences are considered. The larger T-lineage estimate is best viewed as a signal for closer study, not as proof of a lineage-specific age mechanism, particularly because the oldest T-lineage subgroup was small. Attenuation after adding treatment records is also informative: part of the age association may be linked to treatment eligibility, treatment delivery, or clinical frailty, but SEER cannot separate these possibilities. Overall, these analyses support using age as one component of risk discussion rather than as a stand-alone rule for treatment selection.

Social and care-related factors also complicate interpretation of age. Johnston and colleagues found income-related survival differences in SEER, whereas race and income were not associated with OS in their single-center cohort (30). In an NCDB study of adults aged >=40, facility volume and type were not associated with survival after adjustment, although sociodemographic associations remained (31). A SEER study also reported that survival gains differed within the adolescent and young-adult age range (10). These findings caution against treating chronological age as a complete explanation for outcome differences.

Clinically, the results reinforce the need to assess age together with disease characteristics, response, comorbidity, and functional status, as recommended in adult ALL guidance (3, 4, 6, 7). They cannot determine treatment intensity or compare specific therapeutic strategies. The high ALL/LBL-attributable cumulative incidence in older adults is a reason to study how disease control and tolerability can be improved together, rather than an estimate of the benefit of any particular intervention.

The most important limitation is the lack of detailed biological and treatment information. MRD is central to risk assessment (3, 32), but this export lacks MRD, comprehensive cytogenetics, molecular alterations, comorbidity, performance status, treatment intensity, transplantation, and targeted or cellular therapy exposure. Although code 9812 identifies a registry BCR-ABL1 category, NOS does not establish molecular negativity. Changes in coding and ascertainment over 2010–2023 may also affect subtype composition. The selected code set, first-primary restriction, and exclusion of unusable survival records limit generalizability to all patients with ALL/LBL.

Several statistical limitations also remain. Cox PH was violated, and one exploratory split cannot capture every time-varying association. Fine-Gray age proportionality was also not supported for ALL/LBL-attributable death. The log-time test assesses one functional form, while period-specific censoring estimates do not eliminate informative censoring within periods or across other covariates. Subgroup and interaction analyses were exploratory and uncorrected for multiplicity. The spline approximates ages >=85 as 87 and should be interpreted cautiously at its upper end. Fixed age groups should be read as clinically interpretable categories, not as validated biological thresholds.

Cause-of-death attribution may be inaccurate, and censoring 106 unknown-COD deaths cannot determine their true causes. Unknown covariate categories and incomplete treatment records can leave residual bias. Finally, follow-up is short in the latest cohort. A common 3-year horizon creates a more comparable time window, but it does not correct unmeasured case mix, establish equivalent survival, or support 5-year comparisons with 2020-2023.

In this registry cohort, age-related mortality differences were largest during the first year, and ALL/LBL-attributable death predominated by registry attribution even among patients aged >=75. The recent 3-year RMST contrast remained uncertain. Continuous-age and subtype analyses revealed additional heterogeneity, but clinical application will require data with more complete biological, treatment, and functional characterization.

## Data Availability

The original contributions presented in the study are included in the article/**Supplementary Material**. Further inquiries can be directed to the corresponding author.
