# Supplementary material for: Age-stratified survival and competing mortality in adult acute lymphoblastic leukemia and lymphoblastic lymphoma: a population-based competing-risk and restricted mean survival analysis
Source: Front Oncol. 2026 Oct 12;16:1913564. doi: 10.3389/fonc.2026.1913564 (PMC13696205; doi:10.3389/fonc.2026.1913564)
Supplement: Supplementary file 1 [file Table1.docx]

Adult ALL/LBL SEER cohort, 2010-2023; primary cohort N=9,491, excluding Burkitt (9826). Age comparisons use 18-39 years as the reference. P values are unadjusted for multiplicity.

# **Supplementary Table S1. Proportional-hazards diagnostics**

| **Variable** | **Chi-square** | **df** | **P value** |
| --- | --- | --- | --- |
| Age strata | 100.495 | 3 | 1.22e-21 |
| Sex | 4.899 | 1 | 0.0269 |
| Race | 4.107 | 4 | 0.392 |
| Marital status | 23.932 | 3 | 2.58e-5 |
| Diagnosis period | 6.076 | 2 | 0.0479 |
| Histologic subtype | 13.596 | 3 | 0.00351 |
| Global test | 134.264 | 16 | 9.51e-21 |

Scaled Schoenfeld-residual tests used cox.zph(transform="km", terms=TRUE, singledf=FALSE) for the primary Cox model containing age strata, sex, race, marital status, diagnosis period, and histologic subtype, without treatment. Multi-category variables were tested jointly. P<0.05 indicates evidence against PH; P>=0.05 does not prove PH. N=9,491; deaths=4,618.

# **Supplementary Table S2. Cohort flow**

| **Sequential step** | **Retained N** | **Excluded at step** |
| --- | --- | --- |
| Raw exported records | 9844 | 0 |
| Mappable adult age (>=18 years) | 9844 | 0 |
| First primary only | 9844 | 0 |
| Exclude complete dates with 0 days of survival | 9812 | 32 |
| Available nonnegative survival time | 9743 | 69 |
| Diagnosis during 2010-2023 | 9743 | 0 |
| Exclude Burkitt (9826) | 9491 | 252 |

The 69-record exclusion concerns records lacking a usable nonnegative survival time, not to diagnosis-period or age-stratum missingness. All 9,743 records before Burkitt exclusion were within the eligible diagnosis period; 252 Burkitt records were then excluded.

# **Supplementary Table S3. Diagnosis-period RMST and case-mix analyses**

## **S3A. Period-specific estimates and follow-up support**

| **Period** | **N** | **RMST, years** | **SE** | **95% CI** | **At risk at 3 years** |
| --- | --- | --- | --- | --- | --- |
| 2010-2014 | 3077 | 1.89081 | 0.02170 | 1.84829 to 1.93334 | 1406 |
| 2015-2019 | 3439 | 2.09724 | 0.01976 | 2.05851 to 2.13597 | 1865 |
| 2020-2023 | 2975 | 2.14118 | 0.02324 | 2.09563 to 2.18673 | 376 |

## **S3B. Pairwise newer-minus-reference differences**

| **Comparison** | **Difference, years** | **SE difference** | **95% CI** | **P value** |
| --- | --- | --- | --- | --- |
| 2015-2019 vs 2010-2014 | 0.20643 | 0.02935 | 0.14891 to 0.26395 | 2.01e-12 |
| 2020-2023 vs 2010-2014 | 0.25037 | 0.03180 | 0.18805 to 0.31269 | 3.43e-15 |
| 2020-2023 vs 2015-2019 | 0.04394 | 0.03051 | -0.01585 to 0.10373 | 0.150 |

Unadjusted Kaplan-Meier integration to tau=3 years using survival::survfit and summary(fit, rmean=3). Greenwood-based integrated variance supplies each SE. For disjoint period cohorts, SE_difference=sqrt(SE_newer^2+SE_reference^2); 95% CI=difference +/- 1.96*SE_difference; P=2*Phi(-abs(difference/SE_difference)). No bootstrap or multiplicity correction was used. Estimates assume independent censoring within periods and do not adjust for case mix. Numbers at risk include observations at exactly 3 years. Maximum observed follow-up was 13.917, 8.917, and 3.917 years, respectively. The recent-period contrast is not evidence of equivalence.

## **S3C. Reverse Kaplan-Meier follow-up**

| **Period** | **N** | **Median follow-up, months** | **95% CI** |
| --- | --- | --- | --- |
| Overall | 9491 | 66 | 64-68 |
| 2010-2014 | 3077 | 131 | 129-133 |
| 2015-2019 | 3439 | 73 | 71-75 |
| 2020-2023 | 2975 | 22 | 21-23 |

S3D. Two-year RMST sensitivity: period-specific estimates

| Period | N | RMST, years | SE | 95% CI | At risk at 2 years |
| --- | --- | --- | --- | --- | --- |
| 2010-2014 | 3077 | 1.390 | 0.014 | 1.363 to 1.417 | 1612 |
| 2015-2019 | 3439 | 1.510 | 0.012 | 1.486 to 1.535 | 2094 |
| 2020-2023 | 2975 | 1.529 | 0.014 | 1.501 to 1.556 | 903 |

S3E. Two-year RMST sensitivity: pairwise newer-minus-reference differences

| Comparison | Difference, years | SE difference | 95% CI | P value |
| --- | --- | --- | --- | --- |
| 2015-2019 vs 2010-2014 | 0.121 | 0.019 | 0.085 to 0.157 | 7.12e-11 |
| 2020-2023 vs 2010-2014 | 0.139 | 0.020 | 0.101 to 0.178 | 1.41e-12 |
| 2020-2023 vs 2015-2019 | 0.019 | 0.019 | -0.018 to 0.055 | 0.322 |

The 2-year analysis was added as a sensitivity check because the 2020-2023 cohort had more patients at risk at 2 years than at 3 years. It is unadjusted and uses the same Kaplan-Meier RMST approach as S3A-S3B.

S3F. Case-mix distribution by diagnosis period

| Variable | Category | 2010-2014 | 2015-2019 | 2020-2023 | Max pairwise SMD |
| --- | --- | --- | --- | --- | --- |
| Age group | 18-39 | 1253 (40.7) | 1469 (42.7) | 1188 (39.9) | 0.057 |
| Age group | 40-59 | 972 (31.6) | 987 (28.7) | 884 (29.7) | 0.063 |
| Age group | 60-74 | 575 (18.7) | 685 (19.9) | 656 (22.1) | 0.084 |
| Age group | >=75 | 277 (9.0) | 298 (8.7) | 247 (8.3) | 0.025 |
| Sex | Male | 1784 (58.0) | 2009 (58.4) | 1671 (56.2) | 0.045 |
| Sex | Female | 1293 (42.0) | 1430 (41.6) | 1304 (43.8) | 0.045 |
| Race | American Indian/Alaska Native | 39 (1.3) | 53 (1.5) | 44 (1.5) | 0.023 |
| Race | Asian or Pacific Islander | 267 (8.7) | 353 (10.3) | 293 (9.8) | 0.054 |
| Race | Black | 238 (7.7) | 267 (7.8) | 240 (8.1) | 0.012 |
| Race | Unknown | 13 (0.4) | 35 (1.0) | 40 (1.3) | 0.099 |
| Race | White | 2520 (81.9) | 2731 (79.4) | 2358 (79.3) | 0.067 |
| Marital status | Married | 1463 (47.5) | 1546 (45.0) | 1383 (46.5) | 0.052 |
| Marital status | Single | 1139 (37.0) | 1341 (39.0) | 1123 (37.7) | 0.041 |
| Marital status | Divorced/Sep/Widowed | 346 (11.2) | 389 (11.3) | 343 (11.5) | 0.009 |
| Marital status | Unknown | 129 (4.2) | 163 (4.7) | 126 (4.2) | 0.027 |
| Histologic subtype | B-ALL/LBL with BCR-ABL1 | 189 (6.1) | 348 (10.1) | 396 (13.3) | 0.244 |
| Histologic subtype | B-ALL/LBL, NOS | 2262 (73.5) | 2338 (68.0) | 1914 (64.3) | 0.199 |
| Histologic subtype | B-ALL/LBL, other genetic subtypes | 51 (1.7) | 139 (4.0) | 190 (6.4) | 0.242 |
| Histologic subtype | T-ALL/LBL | 575 (18.7) | 614 (17.9) | 475 (16.0) | 0.072 |

Maximum pairwise SMD was calculated across the three diagnosis periods for each category indicator. This descriptive table is not adjusted for outcome and does not make the RMST comparison covariate-adjusted.

Reverse Kaplan-Meier reverses the OS event indicator: last-contact censoring is treated as the event and death as censoring. These estimates describe follow-up, not median OS.

# **Supplementary Table S4. Sensitivity analyses**

| **Analysis** | **Age, years** | **N** | **HR** | **95% CI** | **P value** |
| --- | --- | --- | --- | --- | --- |
| Main cohort (no treatment adjustment) | 40-59 | 9491 | 1.792 | 1.648-1.949 | 2.36e-42 |
| Main cohort (no treatment adjustment) | 60-74 | 9491 | 2.910 | 2.661-3.182 | 3.09e-121 |
| Main cohort (no treatment adjustment) | >=75 | 9491 | 6.258 | 5.627-6.960 | 1.13e-250 |
| Restrict to 2010-2019 | 40-59 | 6516 | 1.748 | 1.593-1.918 | 4.63e-32 |
| Restrict to 2010-2019 | 60-74 | 6516 | 2.854 | 2.582-3.155 | 3.10e-93 |
| Restrict to 2010-2019 | >=75 | 6516 | 6.247 | 5.540-7.045 | 3.41e-196 |
| Exclude unknown race/marital status | 40-59 | 9003 | 1.792 | 1.645-1.953 | 2.65e-40 |
| Exclude unknown race/marital status | 60-74 | 9003 | 2.941 | 2.683-3.224 | 2.90e-117 |
| Exclude unknown race/marital status | >=75 | 9003 | 6.404 | 5.737-7.148 | 3.93e-240 |
| Exclude rare histologic subtypes (other-genetic only) | 40-59 | 9111 | 1.783 | 1.638-1.942 | 1.62e-40 |
| Exclude rare histologic subtypes (other-genetic only) | 60-74 | 9111 | 2.902 | 2.650-3.179 | 9.13e-117 |
| Exclude rare histologic subtypes (other-genetic only) | >=75 | 9111 | 6.240 | 5.600-6.952 | 9.10e-242 |
| Re-include Burkitt (broader code set) | 40-59 | 9743 | 1.802 | 1.660-1.957 | 1.43e-44 |
| Re-include Burkitt (broader code set) | 60-74 | 9743 | 2.877 | 2.634-3.143 | 9.00e-122 |
| Re-include Burkitt (broader code set) | >=75 | 9743 | 6.394 | 5.761-7.096 | 6.90e-267 |
| Exclude 85+ top-coded | 40-59 | 9284 | 1.800 | 1.655-1.958 | 8.59e-43 |
| Exclude 85+ top-coded | 60-74 | 9284 | 2.932 | 2.680-3.207 | 5.70e-122 |
| Exclude 85+ top-coded | >=75 | 9284 | 5.660 | 5.042-6.353 | 6.32e-190 |
| Treatment-adjusted (chemo + radio) | 40-59 | 9491 | 1.738 | 1.598-1.889 | 3.67e-38 |
| Treatment-adjusted (chemo + radio) | 60-74 | 9491 | 2.499 | 2.282-2.737 | 1.22e-86 |
| Treatment-adjusted (chemo + radio) | >=75 | 9491 | 4.473 | 3.988-5.017 | 2.04e-144 |

Models adjust for age strata, sex, race, marital status, diagnosis period, and histologic subtype. The Burkitt re-inclusion analysis uses the broader cohort. Treatment adjustment is a sensitivity analysis only; chemotherapy and radiation are added only in the treatment-adjusted model. Radiation is coded as documented administration versus none/unknown, including refusals and recommendations with uncertain administration. Analyses used R version 4.4.1. Conventional HRs are summary associations under non-PH. The age reference is 18-39 years in every analysis.

# **Supplementary Table S5. Exploratory time-split overall-survival Cox model**

| **Interval** | **Age, years** | **HR** | **95% CI** | **P value** |
| --- | --- | --- | --- | --- |
| 0-1 year | 40-59 | 1.899 | 1.693-2.129 | 5.70e-28 |
| 0-1 year | 60-74 | 3.392 | 3.020-3.810 | 1.70e-94 |
| 0-1 year | >=75 | 8.201 | 7.224-9.311 | 1.22e-231 |
| >1 year | 40-59 | 1.720 | 1.538-1.925 | 2.55e-21 |
| >1 year | 60-74 | 2.457 | 2.169-2.783 | 2.71e-45 |
| >1 year | >=75 | 3.496 | 2.885-4.236 | 2.31e-37 |

Joint age-by-time interaction: likelihood-ratio chi-square=72.193, df=3, P=1.45e-15. Adjustment matches the primary overall-survival model. The 1-year split is exploratory. Zero-month survival was assigned 1/365.25 years only for counting-process time splitting. Other covariates retain constant coefficients; these interval summaries do not establish PH within each interval.

# **Supplementary Table S6. Lineage-stratified age associations**

| **Lineage** | **Age, years** | **N** | **HR** | **95% CI** | **P value** |
| --- | --- | --- | --- | --- | --- |
| B | 40-59 | 7827 | 1.718 | 1.568-1.883 | 5.87e-31 |
| B | 60-74 | 7827 | 2.786 | 2.530-3.068 | 2.80e-96 |
| B | >=75 | 7827 | 5.778 | 5.150-6.482 | 2.74e-196 |
| T | 40-59 | 1664 | 2.135 | 1.736-2.625 | 6.36e-13 |
| T | 60-74 | 1664 | 3.553 | 2.796-4.516 | 3.47e-25 |
| T | >=75 | 1664 | 10.221 | 7.772-13.440 | 3.78e-62 |

B-lineage comprises codes 9811-9819 (n=7,827); T-lineage comprises 9837 (n=1,664). Both models adjust for sex, race, marital status, and period; the B-lineage model additionally adjusts for its constituent histologic subtypes. T-lineage and the T-ALL/LBL subgroup in Table 4 use the same patients and covariates and yield identical estimates. The B-lineage estimate is distinct from B-ALL/LBL NOS.

# **Supplementary Table S7. Exploratory cause-specific Cox analyses**

All models use N=9,491 and the same adjustment as Table 3: age strata, sex, race (White, Black, other/unknown), marital status (married, single, other/unknown), diagnosis period, and histologic group (B-ALL/LBL NOS, genetically defined B-ALL/LBL, T-ALL/LBL). B-ALL/LBL NOS is the histologic reference. Efron ties were used; treatment was not included. Competing deaths were censored at their observed times. Coding A has 3,828 ALL/LBL-attributable and 790 other/unknown-COD events. Coding B censors 106 unknown-COD deaths, leaving 3,828 ALL/LBL-attributable and 684 known other-cause events. Coding B is an alternative assumption about unknown causes.

## **S7A. Summary HRs. Coding A: ALL/LBL-attributable death**

| **Comparison** | **HR** | **95% CI** | **P value** |
| --- | --- | --- | --- |
| Age 40-59 vs 18-39 years | 1.733 | 1.583-1.898 | 1.77e-32 |
| Age 60-74 vs 18-39 years | 2.659 | 2.412-2.932 | 1.46e-85 |
| Age >=75 vs 18-39 years | 5.833 | 5.197-6.547 | 7.60e-197 |
| Female vs male | 0.927 | 0.868-0.989 | 0.0222 |
| Black vs White | 1.147 | 1.021-1.289 | 0.0211 |
| Other/unknown race vs White | 0.812 | 0.729-0.904 | 1.50e-4 |
| Single vs married | 1.089 | 1.002-1.182 | 0.0436 |
| Other/unknown marital status vs married | 1.199 | 1.100-1.308 | 3.57e-5 |
| 2015-2019 vs 2010-2014 | 0.755 | 0.703-0.811 | 1.41e-14 |
| 2020-2023 vs 2010-2014 | 0.668 | 0.611-0.729 | 3.29e-19 |
| Genetically defined B-ALL/LBL vs NOS | 0.775 | 0.700-0.858 | 8.76e-7 |
| T-ALL/LBL vs B-ALL/LBL NOS | 0.887 | 0.810-0.971 | 0.00969 |

Events=3828. HRs are summary associations, not fixed-time risk ratios; age showed evidence of non-PH. See the diagnostics and interval estimates below.

## **S7A. Summary HRs. Coding A: Other/unknown-COD death**

| **Comparison** | **HR** | **95% CI** | **P value** |
| --- | --- | --- | --- |
| Age 40-59 vs 18-39 years | 2.167 | 1.749-2.684 | 1.43e-12 |
| Age 60-74 vs 18-39 years | 4.586 | 3.685-5.708 | 2.06e-42 |
| Age >=75 vs 18-39 years | 9.032 | 6.935-11.764 | 6.35e-60 |
| Female vs male | 0.756 | 0.654-0.874 | 1.59e-4 |
| Black vs White | 1.619 | 1.284-2.041 | 4.52e-5 |
| Other/unknown race vs White | 0.960 | 0.767-1.203 | 0.725 |
| Single vs married | 1.166 | 0.968-1.404 | 0.106 |
| Other/unknown marital status vs married | 1.307 | 1.088-1.571 | 0.00426 |
| 2015-2019 vs 2010-2014 | 0.907 | 0.770-1.068 | 0.241 |
| 2020-2023 vs 2010-2014 | 0.980 | 0.804-1.195 | 0.844 |
| Genetically defined B-ALL/LBL vs NOS | 0.825 | 0.667-1.021 | 0.0770 |
| T-ALL/LBL vs B-ALL/LBL NOS | 0.744 | 0.602-0.919 | 0.00621 |

Events=790. HRs are summary associations, not fixed-time risk ratios; age showed evidence of non-PH. See the diagnostics and interval estimates below.

## **S7A. Summary HRs. Coding B: ALL/LBL-attributable death**

| **Comparison** | **HR** | **95% CI** | **P value** |
| --- | --- | --- | --- |
| Age 40-59 vs 18-39 years | 1.733 | 1.583-1.898 | 1.77e-32 |
| Age 60-74 vs 18-39 years | 2.659 | 2.412-2.932 | 1.46e-85 |
| Age >=75 vs 18-39 years | 5.833 | 5.197-6.547 | 7.60e-197 |
| Female vs male | 0.927 | 0.868-0.989 | 0.0222 |
| Black vs White | 1.147 | 1.021-1.289 | 0.0211 |
| Other/unknown race vs White | 0.812 | 0.729-0.904 | 1.50e-4 |
| Single vs married | 1.089 | 1.002-1.182 | 0.0436 |
| Other/unknown marital status vs married | 1.199 | 1.100-1.308 | 3.57e-5 |
| 2015-2019 vs 2010-2014 | 0.755 | 0.703-0.811 | 1.41e-14 |
| 2020-2023 vs 2010-2014 | 0.668 | 0.611-0.729 | 3.29e-19 |
| Genetically defined B-ALL/LBL vs NOS | 0.775 | 0.700-0.858 | 8.76e-7 |
| T-ALL/LBL vs B-ALL/LBL NOS | 0.887 | 0.810-0.971 | 0.00969 |

Events=3828. HRs are summary associations, not fixed-time risk ratios; age showed evidence of non-PH. See the diagnostics and interval estimates below.

## **S7A. Summary HRs. Coding B: Other-cause death**

| **Comparison** | **HR** | **95% CI** | **P value** |
| --- | --- | --- | --- |
| Age 40-59 vs 18-39 years | 2.362 | 1.866-2.988 | 8.12e-13 |
| Age 60-74 vs 18-39 years | 5.131 | 4.039-6.517 | 6.17e-41 |
| Age >=75 vs 18-39 years | 10.340 | 7.776-13.750 | 4.51e-58 |
| Female vs male | 0.769 | 0.659-0.899 | 9.49e-4 |
| Black vs White | 1.713 | 1.342-2.186 | 1.57e-5 |
| Other/unknown race vs White | 0.925 | 0.723-1.184 | 0.537 |
| Single vs married | 1.172 | 0.959-1.433 | 0.121 |
| Other/unknown marital status vs married | 1.314 | 1.081-1.598 | 0.00613 |
| 2015-2019 vs 2010-2014 | 0.896 | 0.750-1.070 | 0.225 |
| 2020-2023 vs 2010-2014 | 1.018 | 0.824-1.258 | 0.867 |
| Genetically defined B-ALL/LBL vs NOS | 0.860 | 0.688-1.076 | 0.188 |
| T-ALL/LBL vs B-ALL/LBL NOS | 0.688 | 0.543-0.871 | 0.00187 |

Events=684. HRs are summary associations, not fixed-time risk ratios; age showed evidence of non-PH. See the diagnostics and interval estimates below.

## **S7B. PH diagnostics**

### **Coding A: ALL/LBL-attributable death**

| **Variable** | **Chi-square** | **df** | **P value** |
| --- | --- | --- | --- |
| Age strata | 162.741 | 3 | 4.69e-35 |
| Sex | 3.687 | 1 | 0.0548 |
| Race | 4.965 | 2 | 0.0835 |
| Marital status | 37.384 | 2 | 7.62e-9 |
| Diagnosis period | 4.080 | 2 | 0.130 |
| Histologic group | 6.566 | 2 | 0.0375 |
| Global test | 190.772 | 12 | 2.60e-34 |

### **Coding A: Other/unknown-COD death**

| **Variable** | **Chi-square** | **df** | **P value** |
| --- | --- | --- | --- |
| Age strata | 11.228 | 3 | 0.0106 |
| Sex | 0.390 | 1 | 0.532 |
| Race | 2.153 | 2 | 0.341 |
| Marital status | 3.293 | 2 | 0.193 |
| Diagnosis period | 4.703 | 2 | 0.0952 |
| Histologic group | 3.521 | 2 | 0.172 |
| Global test | 23.639 | 12 | 0.0228 |

### **Coding B: ALL/LBL-attributable death**

| **Variable** | **Chi-square** | **df** | **P value** |
| --- | --- | --- | --- |
| Age strata | 162.741 | 3 | 4.69e-35 |
| Sex | 3.687 | 1 | 0.0548 |
| Race | 4.965 | 2 | 0.0835 |
| Marital status | 37.384 | 2 | 7.62e-9 |
| Diagnosis period | 4.080 | 2 | 0.130 |
| Histologic group | 6.566 | 2 | 0.0375 |
| Global test | 190.772 | 12 | 2.60e-34 |

### **Coding B: Other-cause death**

| **Variable** | **Chi-square** | **df** | **P value** |
| --- | --- | --- | --- |
| Age strata | 9.839 | 3 | 0.0200 |
| Sex | 0.533 | 1 | 0.465 |
| Race | 1.268 | 2 | 0.530 |
| Marital status | 2.870 | 2 | 0.238 |
| Diagnosis period | 4.775 | 2 | 0.0919 |
| Histologic group | 4.549 | 2 | 0.103 |
| Global test | 22.779 | 12 | 0.0297 |

## **S7C. Exploratory interval-specific age associations**

### **Coding A: ALL/LBL-attributable death**

| **Interval** | **Age, years** | **HR** | **95% CI** | **P value** |
| --- | --- | --- | --- | --- |
| 0-1 year | 40-59 | 1.906 | 1.684-2.156 | 1.48e-24 |
| >1 year | 40-59 | 1.610 | 1.424-1.820 | 2.99e-14 |
| 0-1 year | 60-74 | 3.339 | 2.945-3.786 | 5.71e-79 |
| >1 year | 60-74 | 2.016 | 1.750-2.322 | 2.37e-22 |
| 0-1 year | >=75 | 8.323 | 7.264-9.537 | 2.35e-204 |
| >1 year | >=75 | 2.406 | 1.897-3.052 | 4.62e-13 |

### **Coding A: Other/unknown-COD death**

| **Interval** | **Age, years** | **HR** | **95% CI** | **P value** |
| --- | --- | --- | --- | --- |
| 0-1 year | 40-59 | 1.864 | 1.376-2.525 | 5.89e-5 |
| >1 year | 40-59 | 2.422 | 1.836-3.194 | 3.72e-10 |
| 0-1 year | 60-74 | 3.760 | 2.788-5.072 | 4.02e-18 |
| >1 year | 60-74 | 5.371 | 4.055-7.115 | 9.75e-32 |
| 0-1 year | >=75 | 7.190 | 5.103-10.130 | 1.68e-29 |
| >1 year | >=75 | 11.327 | 7.905-16.231 | 6.38e-40 |

### **Coding B: ALL/LBL-attributable death**

| **Interval** | **Age, years** | **HR** | **95% CI** | **P value** |
| --- | --- | --- | --- | --- |
| 0-1 year | 40-59 | 1.906 | 1.684-2.156 | 1.48e-24 |
| >1 year | 40-59 | 1.610 | 1.424-1.820 | 2.99e-14 |
| 0-1 year | 60-74 | 3.339 | 2.945-3.786 | 5.71e-79 |
| >1 year | 60-74 | 2.016 | 1.750-2.322 | 2.37e-22 |
| 0-1 year | >=75 | 8.323 | 7.264-9.537 | 2.35e-204 |
| >1 year | >=75 | 2.406 | 1.897-3.052 | 4.62e-13 |

### **Coding B: Other-cause death**

| **Interval** | **Age, years** | **HR** | **95% CI** | **P value** |
| --- | --- | --- | --- | --- |
| 0-1 year | 40-59 | 2.197 | 1.575-3.065 | 3.60e-6 |
| >1 year | 40-59 | 2.474 | 1.822-3.360 | 6.38e-9 |
| 0-1 year | 60-74 | 4.224 | 3.034-5.881 | 1.42e-17 |
| >1 year | 60-74 | 6.001 | 4.420-8.147 | 1.51e-30 |
| 0-1 year | >=75 | 8.549 | 5.901-12.387 | 8.01e-30 |
| >1 year | >=75 | 12.532 | 8.493-18.492 | 3.64e-37 |

## **S7D. Joint age-by-time likelihood-ratio tests**

| **Coding** | **Endpoint** | **Chi-square** | **df** | **P value** |
| --- | --- | --- | --- | --- |
| A | ALL/LBL-attributable death | 114.889 | 3 | 9.72e-25 |
| A | Other-cause death | 4.689 | 3 | 0.196 |
| B | ALL/LBL-attributable death | 114.889 | 3 | 9.72e-25 |
| B | Other-cause death | 3.842 | 3 | 0.279 |

The 1-year split is exploratory and uses the same positive-time convention as S5. Time-varying age terms were fitted jointly with constant coefficients for the other covariates. A nonsignificant age-by-time interaction at this cut-point does not establish PH or contradict a significant Schoenfeld test. These analyses do not fully model non-PH in all covariates.

# **Supplementary Table S8. Additional Fine-Gray checks**

## **S8A. Exploratory age-by-time tests**

| **Endpoint** | **Wald chi-square** | **df** | **P value** |
| --- | --- | --- | --- |
| ALL/LBL-attributable death | 170.223 | 3 | 1.14e-36 |
| Other/unknown-COD death | 4.888 | 3 | 0.180 |

Each primary Fine-Gray model was extended with all three nonreference age indicators multiplied by log(1+t), with time t in years. The three coefficients were jointly tested using their covariance matrix (Wald test, df=3). Baseline age coefficients and other covariates were retained. Coding matches Table 3A; censoring was pooled. The test is exploratory, assesses this time function only, and does not assess proportionality for every covariate. P=0.180 for other/unknown-COD death does not establish proportionality.

## **S8B. Sensitivity to period-specific censoring estimates**

### **ALL/LBL-attributable death**

| **Comparison** | **SHR** | **95% CI** | **P value** |
| --- | --- | --- | --- |
| Age 40-59 vs 18-39 years | 1.657 | 1.515-1.813 | 2.35e-28 |
| Age 60-74 vs 18-39 years | 2.380 | 2.158-2.624 | 1.69e-67 |
| Age >=75 vs 18-39 years | 4.468 | 3.938-5.070 | 3.14e-119 |
| Female vs male | 0.946 | 0.886-1.011 | 0.104 |
| Black vs White | 1.091 | 0.969-1.229 | 0.151 |
| Other/unknown race vs White | 0.828 | 0.742-0.922 | 6.29e-4 |
| Single vs married | 1.070 | 0.985-1.163 | 0.110 |
| Other/unknown marital status vs married | 1.187 | 1.084-1.299 | 2.17e-4 |
| 2015-2019 vs 2010-2014 | 0.767 | 0.713-0.825 | 9.61e-13 |
| 2020-2023 vs 2010-2014 | 0.673 | 0.616-0.736 | 3.96e-18 |
| Genetically defined B-ALL/LBL vs NOS | 0.798 | 0.722-0.882 | 1.01e-5 |
| T-ALL/LBL vs B-ALL/LBL NOS | 0.907 | 0.827-0.994 | 0.0364 |

### **Other/unknown-COD death**

| **Comparison** | **SHR** | **95% CI** | **P value** |
| --- | --- | --- | --- |
| Age 40-59 vs 18-39 years | 1.761 | 1.416-2.191 | 3.66e-7 |
| Age 60-74 vs 18-39 years | 3.014 | 2.414-3.762 | 1.87e-22 |
| Age >=75 vs 18-39 years | 3.377 | 2.601-4.384 | 6.25e-20 |
| Female vs male | 0.798 | 0.690-0.922 | 0.00228 |
| Black vs White | 1.537 | 1.215-1.944 | 3.40e-4 |
| Other/unknown race vs White | 1.034 | 0.827-1.292 | 0.771 |
| Single vs married | 1.083 | 0.894-1.310 | 0.415 |
| Other/unknown marital status vs married | 1.195 | 0.990-1.442 | 0.0636 |
| 2015-2019 vs 2010-2014 | 1.024 | 0.870-1.206 | 0.775 |
| 2020-2023 vs 2010-2014 | 1.126 | 0.922-1.374 | 0.245 |
| Genetically defined B-ALL/LBL vs NOS | 0.905 | 0.731-1.120 | 0.359 |
| T-ALL/LBL vs B-ALL/LBL NOS | 0.761 | 0.616-0.939 | 0.0109 |

These models retain all covariates and primary cause-of-death coding from Table 3 but estimate censoring separately within the three diagnosis periods using cmprsk::crr(cengroup=YearGroup). This addresses between-period censoring differences only; informative censoring within periods and time-varying associations remain possible. The models do not turn calendar-period associations into treatment effects.
